# Comparison of Radiomic Features in a Diverse Cohort of Patients With Pancreatic Ductal Adenocarcinomas

**DOI:** 10.3389/fonc.2021.712950

**Published:** 2021-07-22

**Authors:** Jennifer B. Permuth, Shraddha Vyas, Jiannong Li, Dung-Tsa Chen, Daniel Jeong, Jung W. Choi

**Affiliations:** ^1^ Department of Gastrointestinal Oncology, H. Lee Moffitt Cancer Center & Research Institute, Tampa, FL, United States; ^2^ Department of Cancer Epidemiology, H. Lee Moffitt Cancer Center & Research Institute, Tampa, FL, United States; ^3^ Department of Biostatistics and Bioinformatics, H. Lee Moffitt Cancer Center & Research Institute, Tampa, FL, United States; ^4^ Department of Diagnostic Imaging and Interventional Radiology, H. Lee Moffitt Cancer Center & Research Institute, Tampa, FL, United States

**Keywords:** radiomics, cancer disparities, pancreatic cancer, quantitative imaging, blacks

## Abstract

**Background:**

Significant racial disparities in pancreatic cancer incidence and mortality rates exist, with the highest rates in African Americans compared to Non-Hispanic Whites and Hispanic/Latinx populations. Computer-derived quantitative imaging or “radiomic” features may serve as non-invasive surrogates for underlying biological factors and heterogeneity that characterize pancreatic tumors from African Americans, yet studies are lacking in this area. The objective of this pilot study was to determine if the radiomic tumor profile extracted from pretreatment computed tomography (CT) images differs between African Americans, Non-Hispanic Whites, and Hispanic/Latinx with pancreatic cancer.

**Methods:**

We evaluated a retrospective cohort of 71 pancreatic cancer cases (23 African American, 33 Non-Hispanic White, and 15 Hispanic/Latinx) who underwent pretreatment CT imaging at Moffitt Cancer Center and Research Institute. Whole lesion semi-automated segmentation was performed on each slice of the lesion on all pretreatment venous phase CT exams using Healthmyne Software (Healthmyne, Madison, WI, USA) to generate a volume of interest. To reduce feature dimensionality, 135 highly relevant non-texture and texture features were extracted from each segmented lesion and analyzed for each volume of interest.

**Results:**

Thirty features were identified and significantly associated with race/ethnicity based on Kruskal-Wallis test. Ten of the radiomic features were highly associated with race/ethnicity independent of tumor grade, including sphericity, volumetric mean Hounsfield units (HU), minimum HU, coefficient of variation HU, four gray level texture features, and two wavelet texture features. A radiomic signature summarized by the first principal component partially differentiated African American from non-African American tumors (area underneath the curve = 0.80). Poorer survival among African Americans compared to Non-African Americans was observed for tumors with lower volumetric mean CT [HR: 3.90 (95% CI:1.19–12.78), p=0.024], lower GLCM Avg Column Mean [HR:4.75 (95% CI: 1.44,15.37), p=0.010], and higher GLCM Cluster Tendency [HR:3.36 (95% CI: 1.06–10.68), p=0.040], and associations persisted in volumetric mean CT and GLCM Avg Column after adjustment for key clinicopathologic factors.

**Conclusions:**

This pilot study identified several textural radiomics features associated with poor overall survival among African Americans with PDAC, independent of other prognostic factors such as grade. Our findings suggest that CT radiomic features may serve as surrogates for underlying biological factors and add value in predicting clinical outcomes when integrated with other parameters in ongoing and future studies of cancer health disparities.

## Introduction

Pancreatic cancer is the deadliest malignancy in the United States, with a 5-year relative survival rate of only 10% (1). Due to the lack of effective strategies for prevention, early detection, and treatment, pancreatic cancer is projected to become the second leading cancer killer by 2030 (2). Coinciding with the rise in pancreatic cancer diagnoses and deaths is a notable health disparity, with African Americans/Blacks having significantly higher pancreatic cancer incidence and mortality rates than Non-Hispanic Whites and Hispanic/Latinx (2–12). Biological reasons for these disparities are underexplored and often rely on biomarkers from tissue biopsies, which may not be representative of the entire tumor and its microenvironment. Easily accessible minimally invasive methods that can reflect tumor heterogeneity and correlate with clinical outcomes are urgently needed to advance personalized care for the racially and ethnically diverse population of patients diagnosed with pancreatic cancer each year.

Computed tomography (CT) images are routinely obtained as part of the diagnostic work-up for pancreatic cancer and can be repurposed to support quantitative imaging analyses (13). Radiomics refers to high-throughput extraction and analysis of quantitative features from standard-of-care medical images, many of which are “invisible to the human eye,” to generate mineable data (14). Whereas standard “semantic” radiologic features are typically subjectively and qualitatively measured, computer algorithm-generated radiomic features such as tumor signal intensity, texture, shape, and volume have many advantages (15–21): they represent quantitative, objective measures; reflect tumor heterogeneity and subregional habitats; and are reproducible, stable, and strongly linked to clinical outcomes and underlying molecular data. Radiomic evaluations of pancreas CT scans have been conducted by our team (22–24) and others (15, 25–38), but to date none of these studies have focused on evaluating radiomic features present in pancreatic tumors from AA compared to other ethnic populations. Furthermore, we are unaware of published investigations that specifically compare racial and ethnic differences in radiomic features of different types of non-pancreas tumors. The objective of this study was to compare pretreatment CT radiomic features from a racially and ethnically diverse cohort of cases with pancreatic ductal adenocarcinoma (PDAC), the main histologic subtype of exocrine pancreatic cancers. The implications of this body of work could be far-reaching if radiomic features suggestive of a poor prognosis are identified in the pretreatment setting, in turn influencing clinical decision-making so that more aggressive treatments could be administered earlier to reduce disparities in historically underserved groups.

## Methods

### Study Population

This retrospective cohort was derived from a radiological records database search of individuals with available pretreatment multiphase CT scans and a corresponding histologic diagnosis of PDAC. Cases were diagnosed and treated for PDAC at Moffitt Cancer Center and Research Institute (Tampa, Florida) between 1/2008 and 8/2018. Subjects were excluded if postcontrast venous phase CT imaging was not available or if pathology reports were not available. Race and ethnicity and other covariates were based on self-report. The final analytic dataset included CT images from 71 unique patients (**Table 1**). Ethics approval and written consent to participate were reviewed and approved by Advarra IRB (MCC# 19431; IRB #:Pro00024543).

**Table 1:** Select demographic and clinical characteristics of the pancreatic ductal adenocarcinoma CT radiomic study cohort (N=71).

	AA (n = 23)	H/L (n = 15)	NHW (n = 33)	Overall	P-value
Gender, N (%)					0.993
Female	12 (52.2%)	8 (53.3%)	17 (51.5%)	37 (52.1%)	
Male	11 (47.8%)	7 (46.7%)	16 (48.5%)	34 (47.9%)	
Age at diagnosis, mean (SD)	64.9 (10.2)	61.8 (12.7)	64.9 (10.1)	64.2 (10.6)	0.611
Vital status, N (%)					0.560
Alive	4 (17.4%)	5 (33.3%)	9 (27.3%)	18 (25.4%)	
Dead	19 (82.6%)	10 (66.7%)	24 (72.7%)	53 (74.6%)	
Smoking status, N (%)					0.971
Ever	11 (47.8%)	9 (60.0%)	17 (51.5%)	37 (52.1%)	
Missing	1 (4.35%)	0 (0.00%)	1 (3.03%)	2 (2.82%)	
Never	11 (47.8%)	6 (40.0%)	15 (45.5%)	32 (45.1%)	
Marital status, N (%)					0.516
Divorced	1 (4.35%)	3 (20.0%)	1 (3.03%)	5 (7.04%)	
Married	17 (73.9%)	10 (66.7%)	24 (72.7%)	51 (71.8%)	
Separated	2 (8.70%)	1 (6.67%)	1 (3.03%)	4 (5.63%)	
Single	1 (4.35%)	0 (0.00%)	2 (6.06%)	3 (4.23%)	
Unknown	0 (0.00%)	1 (6.67%)	1 (3.03%)	2 (2.82%)	
Widowed	2 (8.70%)	0 (0.00%)	4 (12.1%)	6 (8.45%)	
Primary site, N (%)					0.265
C241 Ampulla of vater	0 (0.00%)	0 (0.00%)	2 (6.06%)	2 (2.82%)	
C250 Pancreas Head	16 (69.6%)	12 (80.0%)	25 (75.8%)	53 (74.6%)	
C251 Pancreas Body	3 (13.0%)	1 (6.67%)	1 (3.03%)	5 (7.04%)	
C252 Pancreas Tail	0 (0.00%)	2 (13.3%)	4 (12.1%)	6 (8.45%)	
C257 Pancreas Other Specified	1 (4.35%)	0 (0.00%)	0 (0.00%)	1 (1.41%)	
C258 Pancreas Overlapping	1 (4.35%)	0 (0.00%)	1 (3.03%)	2 (2.82%)	
C259 Pancreas, Not otherwise specified	2 (8.70%)	0 (0.00%)	0 (0.00%)	2 (2.82%)	
SEER Derived Stage, N (%)					0.257
Localized	2 (9.09%)	2 (18.2%)	6 (18.2%)	10 (15.2%)	
Regional, by direct extension only	4 (18.2%)	1 (9.09%)	5 (15.2%)	10 (15.2%)	
Regional, to lymph nodes only	4 (18.2%)	0 (0.00%)	0 (0.00%)	4 (6.06%)	
Regional, direct extension and lymph nodes	10 (45.5%)	6 (54.5%)	20 (60.6%)	36 (54.5%)	
Distant	2 (9.09%)	2 (18.2%)	2 (6.06%)	6 (9.09%)	
Tumor grade, N (%)					0.091
Well differentiated	1 (4.6%)	0 (0.0%)	2 (6.01%)	3 (4.35%)	
Moderately differentiated	8 (36.4%)	11 (78.6%)	20 (60.6%)	39 (56.5%)	
Poorly differentiated	6 (27.3%)	3 (21.4%)	8 (24.2%)	17 (24.6%)	
Not determined or Not available	7 (31.8%)	0 (0.0%)	3 (9.1%)	10 (14.5%)	
Clinical tumor size (cm), median (1st ~ 3rd quantile)	3.20 [2.65;4.68]	2.90 [2.40;9.05]	3.65 [2.42;16.0]	3.20 [2.50;9.60]	0.796
Pathological tumor size (cm), median (1st ~ 3rd quantile)	3.0 [2.3;4.7]	3.2 [3.0;4.9]	3.0 [2.5;4.9]	3.0 [2.5;5.2]	0.602
Regional nodes examined, median (1st ~ 3rd quantile)	16.0 [0.0;27.2]	26.0 [21.0;36.5]	17.0 [13.0;22.0]	18.5 [13.0;28.0]	**0.005**
Regional nodes positive, median (1st ~ 3rd quantile)	1.0 [0.5;2.5]	1.0 [0.0;5.5]	1.0 [0.0;2.0]	1.0 [0.0;3.0]	0.840
Survival time (months) median (1st ~ 3rd quantile)	15.0 [9.0;22.5]	24.0 [16.0;27.0]	31.0 [15.0;43.0]	22.0 [13.0;36.0]	**0.028**

AA, African Americans; H/L, Hispanic/Latinx; NHW, Non-Hispanic White; CT, computed tomography; SD, standard deviation; SEER, surveillance, epidemiology, end results program

Some numbers and percentages may not add up to the total due to missing data.

Statistically significant differences are noted in bold font.

### CT Scanner Types, Acquisitions, and Procedures

CT exams were performed on different scanners as represented in **Table 2**, with most scans being performed on a Siemens Sensation 16 (n=31, 43.6%) (Siemens Healthcare, Erlangen, Germany). The post contrast venous phase series was used in this study due to the homogenous availability of this series within our cohort and the superior ability to visualize and segment tumors. The venous phase was generally acquired following weight-based Iopamidol 76% (Bracco Diagnostics Inc., Monroe Township, NJ, USA) dosing to achieve venous phase approximately 60 s post injection. Contrast dosing generally ranged from 75 ml for patients below 55 kg, to 150 ml for patients above 110 kg with gradient increases every 5 kg. Field of view (FOV) ranged from 299 to 500 mm × 299–500 mm based on patient size. The matrix was 512 × 512 for each exam. Slice thickness was 3.0 ± 0.3 mm. Mean venous phase voxel volumes were 1.61, 1.71, and 1.65 mm^3^, for AA, H/L, and NHW, respectively (**Table 2**). At our institution, arterial phase bolus triggering is achieved *via* placement of the contrast tracking region of interest (ROI) over the abdominal aortic lumen at the level of the celiac trunk, with image acquisition triggered at a measured Hounsfield Unit density of 120, and venous phase ensues after a 30 s delay to achieve a 60 s venous phase.

**Table 2:** Scanner type and voxel volumes measured for the study cohort.

	AA (n = 23)	H/L (n = 15)	NHW (n = 33)	P value
Scanner model				0.512
Brilliance 64	1 (4.55%)	0 (0.00%)	0 (0.00%)	
Lightspeed pro 32	1 (4.55%)	1 (6.67%)	3 (9.09%)	
Lightspeed ultra	0 (0.00%)	1 (6.67%)	1 (3.03%)	
Lightspeed VCT	1 (4.55%)	0 (0.00%)	1 (3.03%)	
Sensation 16	13 (59.1%)	5 (33.3%)	13 (39.4%)	
Sensation 40	3 (13.6%)	3 (20.0%)	9 (27.3%)	
Sensation 64	3 (13.6%)	3 (20.0%)	6 (18.2%)	
Somatom definition AS	0 (0.00%)	2 (13.3%)	0 (0.00%)	
Voxel volume (mm^3^), mean (range)	1.6 [1.4;1.8]	1.7 [1.6;2.0]	1.7 [1.4;2.1]	0.303

AA, African Americans; H/L, Hispanic/Latinx; NHW, Non-Hispanic White.

### CT Segmentation and Radiomic Feature Extraction/Reduction

Archived non-contrast and contrast-enhanced CT images were acquired from Moffitt’s GE Centricity Picture Archiving and Communication System (PACS). Our experienced board-certified abdominal oncologic radiologists (JC and DJ) were blinded to patient characteristics and outcomes. For each case, the standardized imaging reporting template for PDAC staging (39) was completed to collect information on “semantic” qualitative-based radiologic features related to morphology, arterial and venous enhancement, and evaluation of extra-pancreatic structures. Whole lesion semi-automated segmentation was performed on each slice of the lesion on all pretreatment (within 3 months prior to treatment) venous phase CT exams using Healthmyne Software (Healthmyne, Madison, WI, USA). The venous phase was chosen in part because this phase was most consistent across all exams. To reduce feature dimensionality, 135 highly relevant non-texture (which measure tumor size, shape, and location) and texture features (which measure properties such as smoothness, coarseness, and regularity) were extracted from each segmented lesion and analyzed for the venous contrast phase. Additionally, CT specifications including scanner type, slice thickness, pixel size were recorded given the known variability that can occur with different scanners and settings (40, 41).

### Statistical Analysis

Data analysis was performed to evaluate racial/ethnic differences in (a) study population characteristics, (b) CT procedures and standard NCCN imaging criteria, and (c) radiomic features. The Kruskal-Wallis test was used for continuous variables, and Chi-squared test or Fisher’s exact test was used for categorical variables to compare the difference among racial/ethnic groups. Significant race/ethnicity-associated radiomic features were determined using a false discovery rate (42) at a threshold of 20%. Spearman correlation analysis was applied to evaluate the correlation between radiomic features. High correlated features were filtered out based on the absolute correlation coefficient above 0.9. Statistically significant radiomic features were summarized by principal component analyses (PCA) to derive a race/ethnicity-associated radiomic signature score as we described previously (43). Receiver operating characteristic (ROC) curve analysis was used to evaluate the prediction efficacy for race/ethnicity using the derived radiomic signature score. Cox proportional hazard regression was performed to evaluate the association between overall survival and each radiomic feature, including interaction terms between median-dichotomized radiomic features and race/ethnicity group (AA *versus* non-AA). Hazards ratios (HR) and 95% confidence intervals (CI) were estimated. Overall survival (OS) was calculated from the date of diagnosis to the date of death or last follow-up using the Kaplan–Meier method. Survival time was censored if patients were lost to follow up or after 4 years. Cox regression analysis was used to identify radiomic features independently prognostic for OS after adjustment for the following clinicopathological variables: age at diagnosis, gender, tumor size, tumor grade, and stage of disease. Statistical tests were two-sided and significant at alpha = 0.05. All statistical analyses were performed using the R 3.6.0 software (https://www.R-project.org).

## Results

### Study Population Characteristics

**Figure 1:**
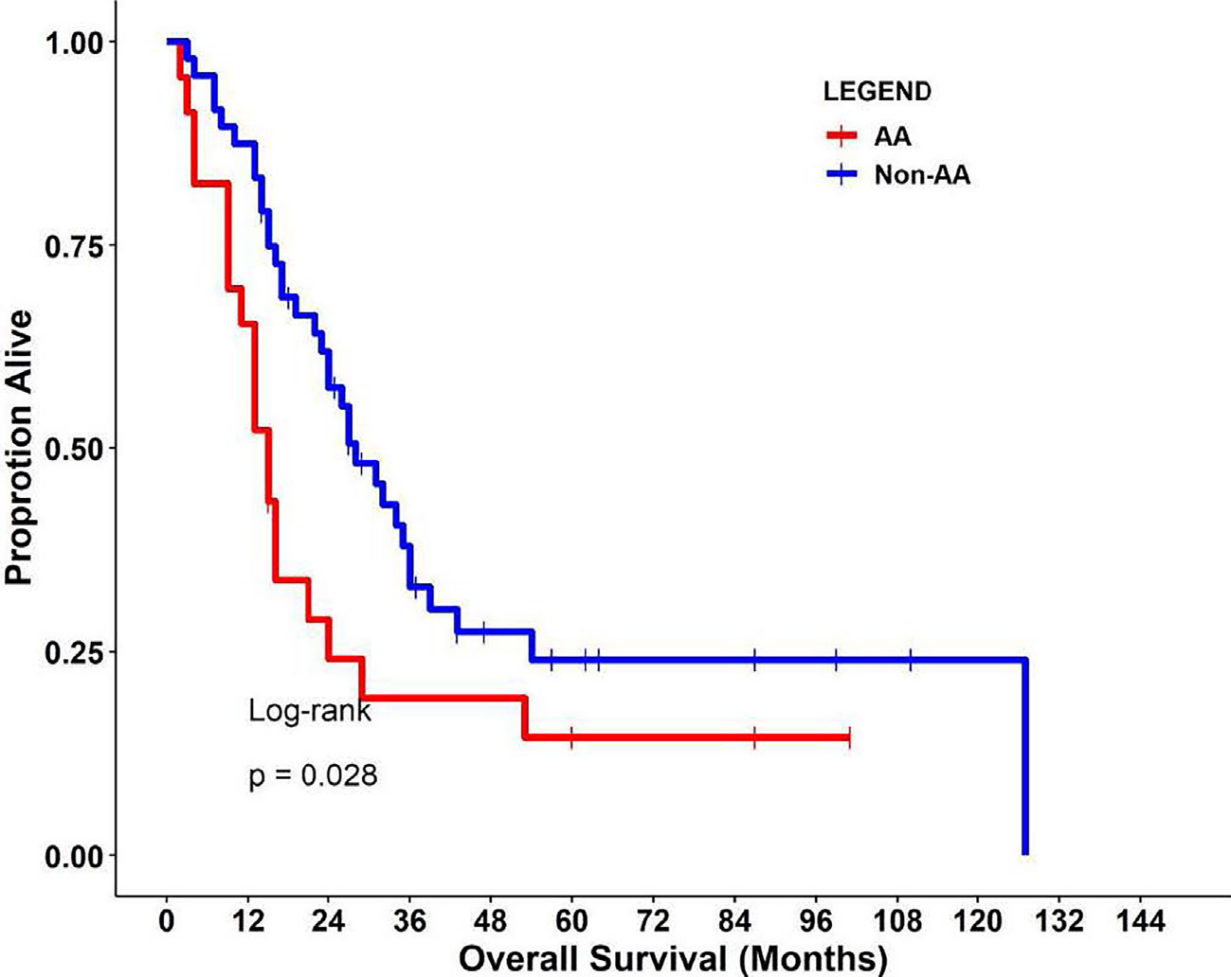
Kaplan-Meier curves (log-rank test) of overall survival in the study cohort.

This retrospective cohort included 71 individuals diagnosed and treated for PDAC at Moffitt Cancer Center and Research Institute (Tampa, Florida) frequency-matched on age-group (+/− 5 years) and gender. Select characteristics of the study population are shown in **Table 1**. There were 23 AA, 15 H/L, and 33 NHW represented, with a slightly higher percentage of females (52%, n=37). The average age at diagnosis was 64.2 years (standard deviation=10.6), and most patients had regional or distant disease. H/L cases had significantly higher numbers of regional nodes examined than AA and NHW (p=0.005), though node positivity was similar between groups (p=0.84). Finally, AA had a significantly shorter average survival time (15 months) compared to Non-AA populations (p=0.028) (**Figure 1**).

### CT Procedures and Standard NCCN Imaging Criteria

No significant differences were observed between racial/ethnic groups in the scanner types used (p=0.512) or in the venous phase voxel volumes (p=0.303) (**Table 2**). Evaluation of standard imaging reporting criteria revealed three parameters that appeared to differ significantly between the three racial/ethnic groups. CT images from the AA group were found to have greater tumor involvement of the superior mesenteric vessels, as measured by degree of superior mesenteric artery (SMA) solid soft tissue contact (p=0.002), extension to the first SMA branch (p=0.036), and superior mesenteric vein (SMV) vessel narrowing and/or contour irregularity (0.033), when compared to NHW (**Table 3**).

**Table 3:** PDAC radiologic reporting template parameters for the study cohort.

Parameter	AA (n = 20)	H/L (n = 14)	NHW (n = 33)	P value
Appearance (vs. parenchyma), N (%)				0.15
Hypodense	18 (90.0%)	11 (78.6%)	22 (66.7%)	
Isodense	2 (10.0%)	3 (21.4%)	11 (33.3%)	
Size (cm) [range]	2.6 [2.0;4.3]	2.6 [2.1;3.0]	2.5[1.8;3.1]	0.734
Location, N (%)				0.385
body/tail	5 (25.0%)	2 (14.3%)	4 (12.1%)	
head/neck	14 (70.0%)	12 (85.7%)	29 (87.9%)	
uncinate	1 (5.0%)	0 (0.0%)	0 (0.0%)	
Pancreatic duct narrowing/abrupt cutoff N (%)	16 (80.0%)	8 (57.1%)	26 (78.8%)	0.244
Biliary tree abrupt cutoff N (%)				0.729
absent	6 (30.0%)	5 (35.7%)	9 (27.3%)	
present	8 (40.0%)	3 (21.4%)	9 (27.3%)	
stent	6 (30.0%)	6 (42.9%)	15 (45.5%)	
*Arterial evaluation*				
*Superior mesenteric artery (SMA) N (%)*				
Solid soft tissue contact	7 (35.0%)	0 (0.0%)	1 (3.0%)	**0.002**
Hazy attenuation/stranding contact	4 (20.0%)	3 (21.4%)	4 (12.1%)	0.232
Focal vessel narrowing or contour irregularity	1 (5.0%)	0	0	0.309
Extension to first SMA branch	6 (30.0%)	2 (14.3%)	2 (6.1%)	**0.036**
*Celiac axis N (%)*				
Solid soft tissue contact	2 (10.0%)	0	0	0.092
Hazy attenuation/stranding contact	2 (10.0%)	1 (7.1%)	1 (3.0%)	0.591
Focal vessel narrowing or contour irregularity	0	0	0	.
*Common Hepatic Artery (CHA) N (%)*				
Solid soft tissue contact	3 (15.0%)	1 (7.1%)	1 (3.0%)	0.246
Hazy attenuation/stranding contact	3 (15.0%)	2 (14.3%)	2 (6.1%)	0.49
Focal vessel narrowing or contour irregularity	1 (5.0%)	0	0	0.309
Extension to celiac axis	1 (5.0%)	0	0	0.309
Extension to bifurcation of hepatic arteries	1 (5.0%)	0	0	0.309
*Arterial variant N (%)*				
Present	3 (15.0%)	3 (21.4%)	3 (9.1%)	0.515
*Venous evaluation*				
*Main portal vein (MPV) N (%)*				
Solid soft tissue contact	7 (35.0%)	2 (14.3%)	9 (27.3%)	0.398
Hazy attenuation/stranding contact	7 (35.0%)	2 (14.3%)	9 (27.3%)	0.398
Focal vessel narrowing or contour irregularity	8 (40.0%)	1 (7.1%)	4 (12.1%)	0.274
*Superior mesenteric vein (SMV) N (%)*				
Solid soft tissue contact	12 (60.0%)	3 (21.4%)	10 (30.3%)	0.055
Hazy attenuation/stranding contact	4 (20.0%)	4 (28.6%)	8 (24.2%)	0.846
Focal vessel narrowing or contour irregularity	8 (40.0%)	1 (7.1%)	5 (15.1%)	**0.033**
*Extrapancreatic evaluation N (%)*				
Liver lesions	3 (15.0%)	2 (14.3%)	3 (9.1%)	0.779
Peritoneal or omental nodules	1 (5.0%)	1 (7.1%)	1 (3.0%)	0.818
Ascites	1 (5.0%)	0	0	0.309
Suspicious lymph nodes	8 (40.0%)	3 (21.4%)	8 (24.2%)	0.385
Venous collaterals	5 (25.0%)	3 (21.4%)	4 (12.1%)	0.465

Possible program errors were observed when contouring inferior margin of mass for one H/L case having a tumor with a cystic component.

Three AA cases also do not have these parameters generated and are not included in this table.

PDAC, pancreatic ductal adenocarcinoma; AA, African American; H/L, Hispanic/Latinx; NHW, non-Hispanic White; cm, centimeters. Bold font indicates a P value < 0.05.

### Radiomic Features

**Figure 2:**
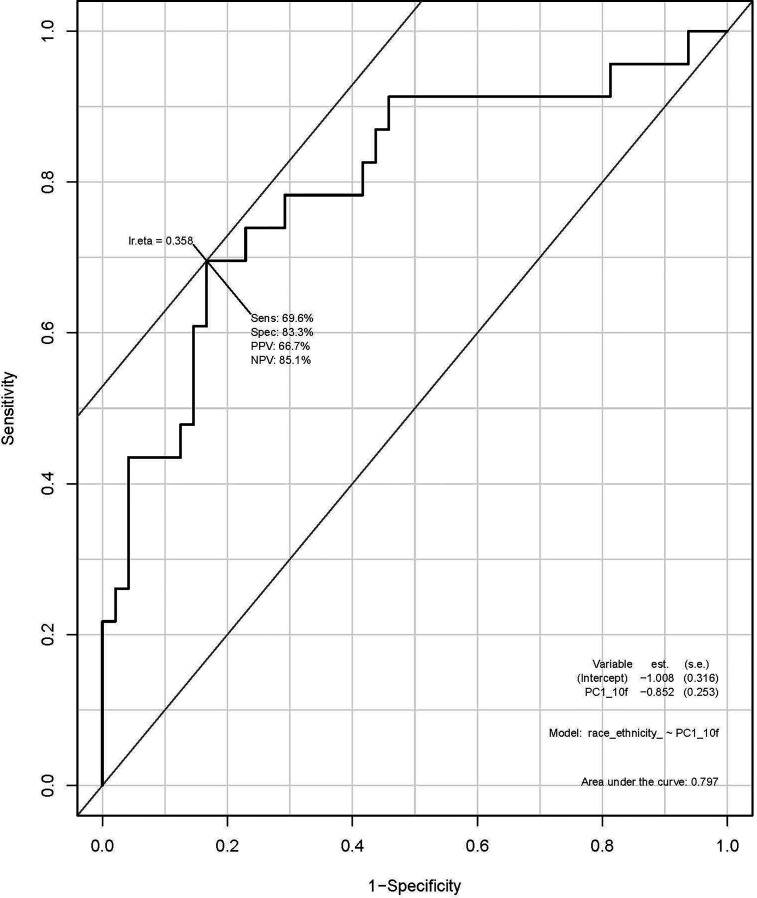
Receiver operating characteristic (ROC) curve using principal component analysis to identify radiomic features predictive of race/ethnicity.

A total of 135 textural and non-textural radiomic features were evaluated for their association with race/ethnicity. Kruskal-Wallis test results indicated that 30 features were significantly associated with race/ethnicity (adjusted p<0.02; **Table 4**). Furthermore, 10 radiomic features were highly associated with race independent of tumor grade and included sphericity, volumetric mean Hounsfield units (HU), minimum HU, coefficient of variation HU, four gray level texture features, and two wavelet texture features (**Supplementary Table 1**). A multivariable model using principal component analysis to represent the radiomic signature yielded an area underneath the curve (AUC)=0.80 in differentiating AA *versus* non-AA (**Figure 2**).

**Table 4:** Radiomic features evaluated in this study and their univariate association with race/ethnicity.

	AA N = 23	H/L N = 15	NHW N = 33	P overall
anterior_posterior_length_mm mean[95%CI]	25.0 [18.0;28.5]	27.0 [17.5;30.0]	25.0 [19.0;28.0]	0.853
asphericity	0.22 [0.17;0.33]	0.22 [0.15;0.26]	0.17 [0.14;0.23]	0.090
coefficient_of_variation	0.34 [0.29;0.44]	0.38 [0.28;0.66]	0.49 [0.41;0.69]	**0.002**
cranial_caudal_length_mm	27.0 [19.0;32.0]	27.0 [21.0;30.5]	24.0 [19.0;35.0]	0.903
elongation	0.87 [0.71;0.93]	0.79 [0.68;0.87]	0.78 [0.68;0.88]	0.413
energy_intensity^2^	5.27 [2.81;10.32]x10^9^	4.30 [2.55;10.39]x10^9^	4.34 [2.51;12.06]x10^9^	0.964
energy_of_ct_number_hu^2^	3.03 [1.41;6.67]x10^7^	3.50 [1.49;6.98]x10^7^	2.56 [0.83;5.51]x10^7^	0.458
entropy_hu	6.70 [6.55;6.90]	6.70 [6.45;7.00]	6.90 [6.70;7.00]	**0.042**
flatness	0.58 [0.51;0.72]	0.62 [0.55;0.69]	0.65 [0.57;0.73]	0.626
glcm_avg_angular_second_moment	0.00 [0.00;0.00]	0.00 [0.00;0.00]	0.00 [0.00;0.00]	.
glcm_avg_column_mean	82.2 [68.0;93.9]	77.1 [46.1;90.7]	65.0 [42.7;81.7]	**0.021**
glcm_avg_column_standard_deviation	30.6 [28.1;40.5]	29.9 [24.7;37.6]	36.7 [31.2;43.9]	**0.037**
glcm_avg_column_var	943 [798;1760]	907 [611;1435]	1370 [973;2113]	0.062
glcm_avg_contrast	1207 [888;1637]	1087 [761;1529]	1580 [1051;2130]	0.055
glcm_avg_correlation	0.38 [0.28;0.42]	0.35 [0.28;0.40]	0.40 [0.34;0.43]	0.325
glcm_avg_dissimilarity	25.4 [22.5;28.8]	25.3 [21.1;28.8]	28.4 [25.2;33.0]	**0.031**
glcm_avg_energy	0.02 [0.02;0.03]	0.02 [0.01;0.03]	0.02 [0.02;0.02]	0.971
glcm_avg_entropy	11.5 [10.7;12.1]	11.5 [10.6;12.1]	11.5 [10.9;12.3]	0.966
glcm_avg_homogeneity	0.04 [0.04;0.05]	0.04 [0.04;0.05]	0.04 [0.03;0.04]	0.067
glcm_avg_row_mean	83.9 [67.2;93.8]	76.7 [40.1;90.5]	66.5 [41.0;79.5]	**0.027**
glcm_avg_row_standard_deviation	27.2 [24.5;30.9]	25.6 [22.5;31.8]	32.2 [26.4;35.2]	**0.014**
glcm_avg_row_var	741 [602;952]	656 [507;1010]	1039 [695;1237]	**0.014**
glcm_cluster_prominence†*	136 [59.5;274]	85.8 [47.0;185]	187 [93.9;368]	**0.035**
glcm_cluster_shade†*	-1.70 [-6.45;-0.05]	-1.40 [-2.55;2.10]	-1.40 [-4.50;6.30]	0.721
glcm_cluster_tendency†*	6.10 [4.20;7.40]	4.90 [3.65;7.75]	7.60 [5.20;8.70]	**0.029**
glcm_contrast†*	1.70 [1.55;2.00]	1.70 [1.30;2.15]	2.10 [1.50;2.80]	**0.040**
glcm_correlation†*	0.50 [0.45;0.60]	0.50 [0.40;0.60]	0.50 [0.50;0.60]	0.374
glcm_difference_average†*	1.00 [0.90;1.05]	1.00 [0.80;1.10]	1.10 [0.90;1.20]	0.096
glcm_difference_entropy†*	1.70 [1.70;1.85]	1.70 [1.60;1.90]	1.80 [1.70;2.00]	0.111
glcm_difference_variance†*	0.70 [0.70;0.90]	0.70 [0.60;0.90]	1.00 [0.70;1.20]	0.027
glcm_dissimilarity†*	1.00 [0.90;1.05]	1.00 [0.80;1.10]	1.10 [0.90;1.20]	0.096
glcm_first_measure_of_information_correlation†*	-0.10 [-0.15;-0.10]	-0.10 [-0.10;-0.10]	-0.10 [-0.10;-0.10]	0.702
glcm_inverse_difference†*	0.60 [0.60;0.60]	0.60 [0.60;0.65]	0.60 [0.60;0.60]	0.084
glcm_inverse_difference_moment†*	0.60 [0.55;0.60]	0.60 [0.50;0.60]	0.60 [0.50;0.60]	0.077
glcm_inverse_difference_moment_normalized†*	1.00 [1.00;1.00]	1.00 [1.00;1.00]	1.00 [1.00;1.00]	.
glcm_inverse_difference_normalized†*	0.90 [0.90;0.90]	0.90 [0.90;0.90]	0.90 [0.90;0.90]	0.717
glcm_inverse_variance†*	0.50 [0.50;0.50]	0.50 [0.50;0.50]	0.50 [0.50;0.50]	0.347
glcm_joint_average†*	6.90 [5.85;8.15]	6.20 [5.90;7.70]	7.30 [6.50;8.30]	0.335
glcm_joint_entropy†*	4.70 [4.50;4.90]	4.60 [4.30;4.95]	5.00 [4.70;5.20]	0.029
glcm_joint_maximum†*	0.10 [0.10;0.10]	0.10 [0.10;0.10]	0.10 [0.10;0.10]	0.331
glcm_joint_variance†*	2.00 [1.55;2.25]	1.60 [1.35;2.40]	2.30 [1.80;2.90]	0.027
glcm_second_measure_of_information_correlation†*	0.60 [0.50;0.70]	0.60 [0.50;0.65]	0.60 [0.50;0.70]	0.470
glcm_sum_average†*	13.7 [11.8;16.3]	12.4 [11.8;15.4]	14.5 [12.9;16.5]	0.347
glcm_sum_entropy†*	3.30 [3.10;3.40]	3.20 [2.95;3.50]	3.50 [3.20;3.60]	0.059
glcm_sum_variance†*	5.20 [3.75;6.40]	4.30 [3.30;7.00]	6.40 [4.80;7.90]	0.045
gldzm_grey_level_nonuniformity	41.6 [25.3;90.2]	44.7 [23.1;83.9]	42.3 [22.0;84.0]	0.827
gldzm_grey_level_nonuniformity_normalised	0.10 [0.10;0.20]	0.20 [0.10;0.20]	0.10 [0.10;0.10]	0.003
gldzm_grey_level_variance	5.60 [5.05;7.60]	6.20 [4.65;8.00]	7.60 [6.60;9.40]	0.012
gldzm_high_grey_level_zone_emphasis	50.5 [42.0;79.0]	48.8 [38.5;63.2]	53.6 [44.3;78.8]	0.442
gldzm_large_distance_emphasis§	2.50 [1.65;3.65]	2.70 [1.80;3.65]	2.70 [1.80;6.10]	0.709
gldzm_large_distance_high_grey_level_emphasis§	107 [71.6;192]	167 [80.7;216]	129 [87.8;267]	0.603
gldzm_large_distance_low_grey_level_emphasis§	0.10 [0.10;0.15]	0.10 [0.10;0.20]	0.10 [0.10;0.20]	0.584
gldzm_low_grey_level_zone_emphasis	0.00 [0.00;0.00]	0.00 [0.00;0.00]	0.00 [0.00;0.00]	0.972
gldzm_small_distance_emphasis§	0.80 [0.70;0.90]	0.80 [0.70;0.90]	0.80 [0.70;0.90]	0.964
gldzm_small_distance_high_grey_level_emphasis§	44.7 [37.1;50.3]	41.1 [25.2;48.7]	43.9 [36.6;65.0]	0.515
gldzm_small_distance_low_grey_level_emphasis§	0.00 [0.00;0.00]	0.00 [0.00;0.00]	0.00 [0.00;0.10]	0.665
gldzm_zone_distance_entropy	3.80 [3.55;4.35]	4.10 [3.60;4.55]	4.20 [3.80;4.60]	0.342
gldzm_zone_distance_nonuniformity	211 [98.3;270]	159 [92.8;272]	180 [120;242]	0.970
gldzm_zone_distance_nonuniformity_normalised	0.60 [0.50;0.70]	0.60 [0.50;0.70]	0.60 [0.40;0.70]	0.924
gldzm_zone_distance_variance	0.60 [0.20;1.05]	0.60 [0.30;1.05]	0.50 [0.30;1.50]	0.867
gldzm_zone_percentage§	0.10 [0.10;0.10]	0.10 [0.00;0.10]	0.10 [0.10;0.10]	0.135
glrlm_grey_level_nonuniformity‡	5986 [2823;10223]	6001 [2708;10739]	6170 [3615;13394]	0.841
glrlm_grey_level_variance‡	2.10 [1.75;2.70]	1.90 [1.50;2.95]	2.90 [2.00;3.40]	0.012
glrlm_high_grey_level_run_emphasis*	46.6 [36.7;68.3]	40.4 [37.1;60.9]	53.9 [43.2;71.9]	0.342
glrlm_normalized_grey_level_nonuniformity‡	0.20 [0.20;0.20]	0.20 [0.20;0.20]	0.20 [0.20;0.20]	0.567
glszm_grey_level_nonuniformity‡	41.6 [25.3;90.2]	44.7 [23.1;83.9]	42.3 [22.0;84.0]	0.827
glszm_grey_level_variance‡	5.60 [5.05;7.60]	6.20 [4.65;8.00]	7.60 [6.60;9.40]	0.012
glszm_high_grey_level_zone_emphasis‡	50.5 [42.0;79.0]	48.8 [38.5;63.2]	53.6 [44.3;78.8]	0.442
glszm_large_zone_emphasis‡	13080 [4011;36509]	12030 [3365;33727]	7602 [2833;30157]	0.742
glszm_large_zone_high_grey_level_emphasis‡	3.89 [1.81;22.75]x10^5^	4.01 [1.81;17.93]x10^5^	3.88 [1.46;12.94]x10^5^	0.884
glszm_large_zone_low_grey_level_emphasis‡	344 [88.8;608]	226 [120;932]	174 [51.7;622]	0.530
glszm_low_grey_level_zone_emphasis‡	0.00 [0.00;0.10]	0.00 [0.00;0.10]	0.00 [0.00;0.10]	0.814
glszm_normalised_zone_size_nonuniformity‡	0.30 [0.30;0.35]	0.30 [0.30;0.40]	0.30 [0.30;0.40]	0.677
glszm_normalized_grey_level_nonuniformity‡	0.10 [0.10;0.20]	0.20 [0.10;0.20]	0.10 [0.10;0.10]	0.003
glszm_small_zone_emphasis‡	0.60 [0.50;0.60]	0.60 [0.60;0.60]	0.60 [0.60;0.60]	0.023
glszm_small_zone_high_grey_level_emphasis‡	29.8 [24.5;46.5]	31.4 [24.7;37.2]	33.1 [27.5;53.3]	0.388
glszm_small_zone_low_grey_level_emphasis‡	0.00 [0.00;0.00]	0.00 [0.00;0.00]	0.00 [0.00;0.00]	0.445
glszm_zone_percentage‡	0.10 [0.10;0.10]	0.10 [0.00;0.10]	0.10 [0.10;0.10]	0.135
glszm_zone_size_entropy‡	5.10 [4.80;5.50]	4.80 [4.65;5.35]	5.30 [5.00;5.50]	0.085
glszm_zone_size_nonuniformity‡	111 [46.7;169]	129 [34.4;172]	108 [63.4;190]	0.981
glszm_zone_size_variance‡	12621 [3911;35884]	11642 [3255;33387]	7458 [2756;29728]	0.742
hu_kurtosis	3.58 [3.24;4.16]	3.34 [3.13;4.08]	3.49 [3.13;4.06]	0.723
hu_skewness	-0.20 [-0.35;0.02]	-0.05 [-0.26;0.13]	-0.10 [-0.30;0.28]	0.486
hu_uniformity	66.2 [56.6;71.3]	62.0 [34.1;72.3]	50.6 [30.6;59.1]	0.002
hu_uniformity_acr	-33.30 [-65.15;-10.45]	-49.20 [-116.95;-1.35]	-85.70 [-117.80;-41.00]	0.061
maximum_ct_number_hu	175 [160;210]	190 [151;238]	181 [159;231]	0.999
median_ct_number_hu	83.0 [67.0;94.5]	78.0 [39.0;90.5]	67.5 [41.0;82.0]	0.045
mesh_compactness_1_mm	20.9 [14.4;31.1]	22.4 [16.5;33.4]	23.9 [16.6;34.2]	0.895
mesh_compactness_2_mm	0.55 [0.43;0.63]	0.56 [0.50;0.67]	0.62 [0.53;0.68]	0.092
mesh_sa_to_volume_ratio	0.32 [0.26;0.42]	0.31 [0.26;0.39]	0.29 [0.24;0.38]	0.804
minimum_ct_number_hu	-30.00 [-57.50;-14.00]	-39.00 [-83.00;-15.00]	-58.00 [-74.00;-46.00]	0.021
ngldm_dependence_count_percentage‡	1.00 [1.00;1.00]	1.00 [1.00;1.00]	1.00 [1.00;1.00]	.
ngldm_dependence_energy‡	0.00 [0.00;0.00]	0.00 [0.00;0.00]	0.00 [0.00;0.00]	0.034
ngldm_dependence_entropy‡	5.80 [5.75;6.15]	5.80 [5.70;6.10]	6.00 [5.80;6.20]	0.212
ngldm_dependence_nonuniformity‡	380 [192;771]	326 [167;675]	331 [187;747]	0.994
ngldm_dependence_variance‡	11.4 [9.85;12.7]	10.1 [9.75;14.6]	10.4 [7.90;12.4]	0.466
ngldm_gl_nonuniformity‡	895 [485;2395]	720 [406;2023]	767 [386;1999]	0.808
ngldm_gl_variance‡	2.00 [1.55;2.45]	1.70 [1.35;2.60]	2.70 [1.80;3.20]	0.013
ngldm_high_dependence_emphasis‡	56.1 [45.9;67.8]	58.8 [48.2;78.6]	51.6 [37.8;62.7]	0.361
ngldm_high_dependence_high_gl_emphasis‡	2250 [1966;3719]	2594 [1677;3588]	2946 [1948;4005]	0.964
ngldm_high_dependence_low_gl_emphasis‡	1.10 [0.85;1.70]	1.50 [0.80;2.40]	1.00 [0.80;1.50]	0.330
ngldm_high_gl_dependence‡	49.9 [36.2;69.2]	39.7 [37.0;62.2]	54.4 [45.4;68.9]	0.360
ngldm_low_dependence_emphasis‡	0.10 [0.10;0.10]	0.10 [0.10;0.10]	0.10 [0.10;0.10]	0.088
ngldm_low_dependence_high_gl_emphasis‡	5.10 [3.45;5.75]	4.40 [2.20;6.40]	5.30 [3.80;8.20]	0.186
ngldm_low_dependence_low_gl_emphasis‡	0.00 [0.00;0.00]	0.00 [0.00;0.00]	0.00 [0.00;0.00]	.
ngldm_low_gl_dependence‡	0.00 [0.00;0.00]	0.00 [0.00;0.00]	0.00 [0.00;0.00]	0.879
ngldm_normalized_dependence_nonuniformity‡	0.10 [0.10;0.10]	0.10 [0.10;0.10]	0.10 [0.10;0.10]	0.034
ngldm_normalized_gl_nonuniformity‡	0.20 [0.20;0.20]	0.20 [0.20;0.30]	0.20 [0.20;0.20]	0.008
rms_of_ct_number_hu	89.6 [71.4;97.0]	83.9 [49.3;94.8]	74.3 [50.5;87.2]	0.067
sphericity	0.82 [0.75;0.86]	0.82 [0.79;0.87]	0.85 [0.81;0.88]	0.084
standard_deviation_of_ct_number_hu	27.2 [24.6;30.9]	25.6 [22.7;31.8]	32.3 [26.4;35.3]	0.014
surface_area_mm^2^	2077 [1241;3104]	2215 [1464;3284]	1771 [1408;3043]	0.879
transverse_length_mm	26.0 [20.0;33.0]	24.0 [22.5;31.5]	25.0 [19.0;29.0]	0.716
volume_mm^3^	5522 [2863;11750]	6748 [3892;13092]	6075 [4018;12569]	0.919
volumetric_length_mm	34.0 [25.0;39.0]	35.0 [26.0;45.0]	31.0 [28.0;38.0]	0.810
volumetric_mean_of_ct_number_hu	83.9 [67.2;93.8]	76.7 [40.1;90.5]	66.5 [41.0;79.5]	0.027
wavelet_hhl_10th_percentile_hu	-4.80 [-5.80;-4.00]	-5.30 [-5.85;-4.70]	-5.2 0[-6.90;-4.30]	0.310
wavelet_hhl_90th_percentile_hu	4.90 [4.10;5.85]	5.30 [4.65;5.80]	5.30 [4.30;7.00]	0.454
wavelet_hhl_coefficient_of_variation	320 [-92.90;753]	114 [-379.00;175]	-80.70 [-454.90;244]	0.163
wavelet_hhl_energy_hu^2^	5.90 [3.08;15.48]x10^4^	1.04 [0.29;1.87]x10^5^	9.86 [4.00;15.98]x10^4^	0.707
wavelet_hhl_entropy	12.0 [11.2;13.3]	11.7 [10.9;13.1]	11.8 [11.1;13.2]	0.980
wavelet_hhl_excess_kurtosis	0.10 [0.00;0.25]	0.20 [0.10;0.40]	0.20 [0.10;0.50]	0.104
wavelet_hhl_interquartile_range_hu	5.10 [4.25;6.10]	5.40 [4.90;5.95]	5.60 [4.70;7.20]	0.466
wavelet_hhl_minimum_hu	-16.20 [-19.05;-12.35]	-19.50 [-23.70;-13.75]	-17.80 [-28.80;-14.40]	0.163
wavelet_hhl_maximum_hu	16.6 [11.6;18.2]	18.5 [15.3;22.7]	18.3 [14.0;26.5]	0.129
wavelet_hhl_mean_deviation_hu	3.00 [2.50;3.60]	3.30 [2.90;3.55]	3.30 [2.70;4.30]	0.321
wavelet_hhl_mean_hu	0.00 [0.00;0.00]	0.00 [0.00;0.00]	0.00 [0.00;0.00]	0.854
wavelet_hhl_median_deviation_hu	3.00 [2.50;3.60]	3.30 [2.90;3.55]	3.30 [2.70;4.30]	0.326
wavelet_hhl_median_hu	0.00 [0.00;0.00]	0.00 [0.00;0.00]	0.00 [0.00;0.10]	0.653
wavelet_hhl_quartile_coefficient_of_dispersion	45.7 [-105.15;289]	34.2 [-81.30;63.6]	63.6 [-66.20;179]	0.315
wavelet_hhl_range_hu	32.4 [24.0;38.0]	39.7 [28.8;50.5]	35.9 [28.3;54.8]	0.157
wavelet_hhl_robust_mean_deviation_hu	2.10 [1.75;2.50]	2.20 [2.00;2.50]	2.30 [1.90;3.00]	0.442
wavelet_hhl_rms_hu	3.80 [3.20;4.60]	4.30 [3.65;4.55]	4.30 [3.40;5.50]	0.254
wavelet_hhl_skeweness	0.00 [0.00;0.00]	0.00 [0.00;0.00]	0.00 [0.00;0.00]	0.419
wavelet_hhl_variance_hu^2^	14.3 [10.2;20.9]	18.3 [13.3;20.6]	18.8 [11.6;30.2]	0.218

Some P values are missing because they were unable to be estimated.

AA, African American; H/L, Hispanic/Latinx; NHW, non-Hispanic White; MM, millimeters; CT, computed tomography; HU, Hounsfield Units; GLCM, gray level cooccurrence matrix; AVG, average; VAR, variance; GLDZM, gray level distance zone matrix; American College of Radiology; SA, surface area; NGLDM, neighborhood gray-level different matrix; GL, gray level; RMS, root mean square; HHL, high-pass high-pass and low-pass filters.

^†^gray leveled image; *ibsi by slice with merging; ^‡^as volume with full merging; ^§^with full merging. Bold font indicates a P value < 0.05.

**Figure 3:**
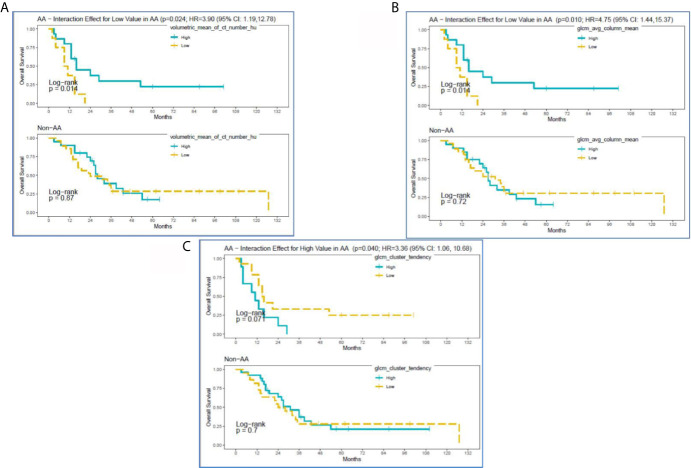
Kaplan-Meier curves for significant interactions between radiomic features and overall survival among self-reported African American (AA) and Non-AA (Hispanic/Latinx, H/L; and Non-Hispanic White, NHW) groups according to **(A)** Volumetric Mean CT (HU), **(B)** GLCM Avg Column Mean, and **(C)** GLCM Cluster Tendency.

Survival analysis identified the following non-correlated radiomic features with a significantly different survival difference between AA and non-AA (interaction effect between radiomic features and race with p<0.05): Volumetric Mean CT (HU) (HR: 3.90 (95% CI:1.19–12.78), p=0.024), GLCM Avg Column Mean (HR:4.75 (95% CI: 1.44,15.37), p=0.010), and GLCM Cluster Tendency (HR:3.36 (95% CI: 1.06–10.68), p=0.040) (**Supplementary Table 2**). Specifically, for Volumetric Mean CT and GLCM Avg Column Mean in tumors, low value of these radiomic features was associated with poorer survival among AA (**Figures 3A, B**). In contrast, survival curves overlapped between low and high groups of the radiomic features among non-AA. As a result, survival differences due to the radiomic features became differential between racial/ethnic groups (p=0.01–0.02). The GLCM Cluster Tendency (**Figure 3C**) had an opposite trend with high values associated with poorer survival among AA, but slightly improved survival among non-AA, leading to a significant differential survival difference between AA and non-AA (p=0.04). Furthermore, multivariate survival analysis indicated that Volumetric Mean CT (HU) and GLCM Avg Column Mean remain significantly associated with OS between AA and non-AA after adjustment for clinical-pathological features including age at diagnosis, gender, tumor size, tumor grade, and SEER-derived stage. Lower values of these radiomic features were associated with worse survival among AA (**Supplementary Table 3**).

**Figure 4:**
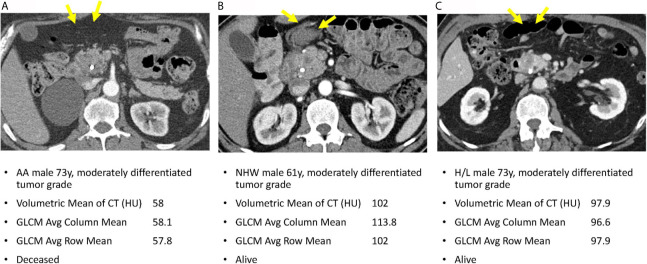
Axial venous phase CT images are presented in PDAC patients matched for tumor grade, gender, and age-group. Image **(A)** from an AA patient and shows a poorly defined hypoenhancing tumor marked by the yellow arrows. Image **(B)** in a NHW shows a similar radiologic appearance of the tumor but with significantly different radiomic tumor values. Image **(C)** in a Hispanic patient also had radiomic values different from the AA case. Note that a common bile duct stent is present in each of these patients.

**Figure 4** reveals pretreatment CT images for three PDAC patients matched on tumor grade, gender, and age-group; lower radiomic values were observed among tumors from AA in volumetric mean CT HU and two GLCM texture features, compared to non-AA. These observations suggest that although the pancreatic tumors may appear similar on CT images, they reflect significantly different radiomic values associated with race/ethnicity and are predictive of overall survival.

## Discussion

We conducted the first investigation we are aware of to apply a radiomic approach to routine pretreatment CT scans from patients with PDAC to specifically explore associations with race/ethnicity and overall survival. Our analysis showed AA patients with low volumetric mean HU tumors had worse survival than similar tumors in non-AA. In PDAC, tumors with HU lower than surrounding pancreatic parenchyma have been correlated with worse outcomes (44). In our study, the low volumetric mean HU may be revealing a similar relationship to survival as the previously reported relative delta score, except that our measure is based in absolute HU as opposed to the delta score, which reflects relative differences in HU. Our analysis also demonstrated worse survival in AA patients having high coefficient of variation HU compared to similar tumors in non-AA, independent of key prognostic factors. The coefficient of variation HU is a reflection of tumor heterogeneity as it presents on CT based on voxel HU values, and it represents the standard deviation of the HU values within segmented tumors divided by the mean HU. Therefore, tumors with a wider range of different-appearing voxels within a tumor will have a larger coefficient of variation HU. In line with these findings, previous studies have shown that more heterogenous tumors are associated with high-grade dysplasia, resistance to anticancer therapies, and poorer prognoses (1, 45–48).

In this study, radiomics allowed us to preoperatively and non-invasively quantify the differences in appearance of pancreatic tumors across different racially and ethnically defined cohorts, even where the differences were not easy to visualize or describe qualitatively. We discovered multiple radiomic features that predict poor survival specifically in AA patients independent of other demographic and clinical factors. It is possible that these radiomic differences reflect inherent biological tumor differences specific to each ethnic group. Having potential poor prognostic biomarkers available in the pretreatment setting could influence clinical decisions and support earlier and more aggressive treatments that could reduce disparities for these underserved groups. Additionally, future studies correlating race/ethnicity-based radiomic features with tumor tissue-based biomarkers are needed to determine the capacity at which radiomics can be used in clinical decision-making workflows at the time of multidisciplinary tumor board.

We realize that the single-institutional retrospective design is prone to biases, but there is wealth in this exploratory investigation. Future prospective multicenter studies involving racially diverse cohorts of PDAC cases will be needed to continue to move PDAC disparities research forward. We plan to optimize and validate the most promising radiomic features and biomarkers in an independent cohort of AA PC cases using our multi-institutional Florida Pancreas Collaborative infrastructure (49). Furthermore, we plan to conduct a radiogenomic approach that integrates CT radiomic data with molecular biomarker data from pancreatic tumor tissue in order to uncover biological mechanisms to explain the disproportionate PDAC burden in AA.

## Data Availability Statement

The original contributions presented in the study are included in the article/**Supplementary Material**. Further inquiries can be directed to the corresponding author.

## Ethics Statement

The studies involving human participants were reviewed and approved by Advarra IRB (MCC# 19431; IRB #: Pro00024543). The patients/participants provided their written informed consent to participate in this study.

## Author Contributions

JP, DJ, and JC contributed to conception and design of the study. SV organized the database. JL and DT-C performed the statistical analysis. JP wrote the first draft of the manuscript, and JL, D-TC, DJ, and JC wrote sections of the manuscript. All authors contributed to the article and approved the submitted version.

## Funding

This research was funded in part through the George Edgecomb Society awarded to JP, JC, DJ, and D-TC. The research was also supported by the Quantitative Imaging Core and the Biostatistics and Bioinformatics Shared Resource at the H. Lee Moffitt Cancer Center & Research Institute, an NCI designated Comprehensive Cancer Center (P30-CA076292).

## Conflict of Interest

The authors declare that the research was conducted in the absence of any commercial or financial relationships that could be construed as a potential conflict of interest.
